# Refuting misconceptions in medical physiology

**DOI:** 10.1186/s12909-020-02166-6

**Published:** 2020-08-05

**Authors:** M. Versteeg, M. H. van Loon, M. Wijnen-Meijer, P. Steendijk

**Affiliations:** 1 Center for Innovation in Medical Education, Leiden University Medical Center, Albinusdreef 2, 2333 ZA Leiden, The Netherlands; 2 Department of Cardiology, Leiden University Medical Center, Leiden, the Netherlands; 3 Department of Developmental Psychology and Swiss Graduate School for Cognition, Learning and Memory, University of Bern, Bern, Switzerland; 4 Technical University of Munich, TUM School of Medicine, TUM Medical Education Center, Munich, Germany

**Keywords:** Refutation text, Misconceptions, Physiology, Conceptual change

## Abstract

**Background:**

In medical physiology, educators and students face a serious challenge termed misconceptions. Misconceptions are incorrect ideas that do not match current scientific views. Accordingly, they have shown to hamper teaching and learning of physiological concepts. Conceptual Change Theory forms the basis of new teaching and learning practices that may alleviate misconceptions and facilitate critical thinking skills that are essential in becoming knowledgeable, self-regulated health professionals. In this study, we examined if such an intervention named refutation texts, could enhance medical students’ cognition and metacognition.

**Methods:**

First-year medical students (*N* = 161) performed a pre-test and post-test on cardiovascular physiology concepts, including a self-perceived confidence rating. In between, students read either a standard text with an explanation of the correct answer, or a refutation text which additionally refuted related misconceptions.

**Results:**

In both groups, average performance scores (refutation: + 22.5%, standard: + 22.8%) and overall confidence ratings (refutation: Δ0.42 out of 5, standard: Δ0.35 out of 5) increased significantly (all *p* < .001), but a significant effect of the specific refutation element was not found. Initially incorrect answers were corrected less frequently in cases of high confidence (35.8%) than low confidence (61.4%).

**Conclusions:**

Our results showed that refutation texts significantly increased students’ knowledge, however, the refutation element did not have a significant additional effect. Furthermore, high confidence in incorrect answers negatively affected the likelihood of correction. These findings provide implications for teaching practices on concept learning, by showing that educators should take into account the key role of metacognition, and the nature of misconceptions.

## Background

Physiology plays a central role in understanding human body functions [1]. It is therefore problematic that many medical students find it difficult to acquire accurate knowledge of physiological concepts [2]. This may be partially due to the presence of misconceptions [3–5]. Misconceptions can be defined as incorrect ideas that do not match current scientific views [6]. Over the last 30 years, misconceptions in science education have been demonstrated repeatedly [7–10]. Misconceptions are resistant to change as they frequently persist even after direct instruction [11]. In medical education this topic is less well studied and few didactic strategies have been put forward to address misconceptions and promote conceptual change among students.

The process of shifting from an incorrect scientific understanding to a correct one is strongly influenced by what a learner already knows [12]. A learner’s prior knowledge should therefore always be engaged while trying to understand new information. Sometimes, the to-be-learned information conflicts with one’s prior knowledge. It then requires reorganization of cognitive schemas in the brain to accommodate novel information [13, 14]. When such cognitive conflicts concern conceptual knowledge, this reorganization process is referred to as conceptual change [15]. By contaminating the learner’s prior knowledge, misconceptions may inhibit rather than facilitate learning of new information [13, 16, 17].

Teachers may not succeed to alleviate misconceptions by simply providing the right answer or explanation to a question [17, 18]. Educational researchers have proposed that teaching strategies should explicitly undermine, i.e. refute, learners’ misconceptions [19], for example by using refutation texts. Refutation texts typically comprise three components: (1) the common misconception, (2) the refutation which explicitly debunks the misconception, (3) the correct answer [18, 19].

A refutation text for the misconception that blood slows down at a vessel narrowing states: *Many people think that the velocity of blood decreases when it enters a constricted section of a vessel, just like cars slow down when the road narrows, (*i.e. *misconception) but this notion is false since blood, being a liquid, cannot be compressed (*i.e. *refutation). The velocity of blood actually increases because the same blood volume has to pass through a smaller cross-section in the same time frame (*i.e. *correct answer).*

The potential of refutation texts to induce conceptual change has been demonstrated in various science domains including physics [19] and biology [20, 21]. Superiority of this instructional approach is presumably based on the mechanism of coactivation as described in the Knowledge Revision Components framework [13]. Coactivation of the misconception and correct concept appears crucial for establishing learners’ awareness of the existing conflict. Knowing that there is an apparent inconsistency between prior knowledge and new information may lead to an experience of cognitive conflict, followed by attempts to resolve this issue [22, 23]. In the case of refutation texts, the cognitive conflict may be induced by reading the common misconception plus a refutation that debunks this misconception. This conflict may lead to the reorganization of cognitive schemas in the brain [13, 14]. Such reorganizations as induced by refutation texts reflect the process of conceptual change, ideally resulting in accurate conceptual understanding.

The cognitive effect of refutation texts on learners’ conceptual understanding depends on a learner’s metacognition. Metacognition is the process of thinking about one’s thinking [24], and encompasses an important component referred to as metacognitive knowledge [25]. Metacognitive knowledge entails being aware of what you do and do not know, e.g. when reading refutation texts this could entail becoming aware of a cognitive conflict [25]. Only students who can accurately judge, i.e. metacognitively evaluate that their understanding of a concept is insufficient may choose to further study this concept [26, 27]. Building on the importance of metacognition in concept learning, refutation texts may stimulate students’ conceptual change through enhancing their metacognitive knowledge [18, 26]. Various studies in elementary school and higher education support this hypothesis [20, 28], however, others failed to demonstrate enhancement of students’ metacognitive knowledge after reading refutation texts [29]. So, despite the theoretical link between conceptual change and metacognitive knowledge, research investigating the influence of refutation texts on learners’ metacognitive knowledge remains limited and so far inconclusive.

In addition to investigating the influence of refutation texts on students’ conceptual understanding and metacognitive knowledge, it may be of interest to examine which learner characteristics are most productive for facilitating conceptual change. Some studies distinguish between misconceptions held by learners with low-confidence versus misconceptions held with high-confidence [29–31]. Of note, in some studies the term misconception is reserved exclusively for the latter type, whereas ‘wrong answers’ held with low confidence are referred to as lack of knowledge. Researchers have suggested that high-confidence misconceptions are hardest to correct because they are more strongly represented in memory and they impair the student in accommodation contrasting information [32, 33]. The hypercorrection effect, however, contradicts this hypothesis, stating that corrective feedback induces coactivation which may particularly surprise learners who are highly confident about their misconceptions, thereby increasing their attention and enhancing text comprehension [34–36].

In this study, we investigated the cognitive effect, metacognitive effect and hypercorrection effect in a refutation text intervention. Firstly, we investigated if reading refutation texts improves actual knowledge (i.e. cognition). Secondly, we studied if reading refutation texts improves self-perceived knowledge (i.e. metacognition). Thirdly, we tested if the hypercorrection effect occurred: we hypothesised that high-confidence incorrect answers would be corrected more frequently than low-confidence incorrect answers. In summary, we presume that research on conceptual change interventions in medical education is needed to improve students’ conceptual understanding. Moreover, equipping students with accurate knowledge about physiological concepts may ultimately result in improved clinical reasoning and decision-making [37, 38].

## Methods

### Participants and setting

This study was conducted in first-year medical students at the Leiden University Medical Center. At the start of the academic year the student cohort was divided into 24 groups, 12 groups (12–15 students/group, total 161 students) were included in this study. The protocol was implemented in a seminar on cardiovascular physiology. This seminar was part of a compulsory, 8-weeks course on integrative cardiovascular, respiratory and kidney physiology at the beginning of the second semester. The course seminars focus on solving clinically-based scenarios in small-group sessions led by an expert. This study was performed during the first course seminar, which focusses on the concepts of flow, pressure and resistance. These concepts were introduced and explained in a plenary lecture a few days before the seminar and the students were instructed to study them prior to the seminar also using a specified section from a medical physiology textbook [39].

This study protocol was approved by the Leiden University Medical Center Educational Research Review Board (ERRB), reference number: OEC/ERRB/20171010/2. Students provided written informed consent to use their responses for scientific analysis and publication. They received no additional credit and they were informed that all data would be anonymised and test performance had no effect on their course grade. They could withdraw their permission at any time.

### Procedure

In our study, half of the groups were assigned to the Refutation text intervention, and the other half received a Standard text control intervention. Allocation to these two experimental groups was arbitrarily except for the aim to have a similar male-to-female ratio (30:70) in all groups. The study was performed in a classroom setting at the beginning of a seminar. All students performed a pre-test, followed by either a Refutation text or Standard text intervention, and a subsequent post-test with near-transfer questions. Both tests were given on paper and consisted of four multiple-choice multi-tier questions (6). All questions were about cardiovascular physiology topics regarding flow, pressure and resistance. In between the pre-test and post-test, each student received either refutation texts or standard texts (see Additional file 1: Appendix A for examples). The standard texts gave, for each question of the pre-test, the right answer plus an explanation (average 177 words/text). Students had four minutes to answer each question on the pre-test and post-test, and also four minutes for reading each refutation or standard text. The study was teacher-paced, meaning that students had to wait for the next question or text if they finished earlier, resulting in a total time of 48 min. The refutation texts groups followed a similar procedure, except that the texts contained an additional sentence (i.e. refutation element) that presented a common misconception with an explicit refutation of that misconception, before providing the correct answer with the explanation (average 226 words/text). During the tests, students received a summary sheet with all the relevant factual knowledge to reduce the number of incorrect answers merely due to lack of factual knowledge.

### Materials

The questions and explanations were designed by a physiology teacher (P.S.) with longstanding experience in cardiovascular research and teaching, and designing and reviewing exam questions. Each question consisted of three tiers, i.e. an answer tier, an explanation tier, and a confidence tier (see Additional file 1: Appendix B for examples). In the answer tier, students were asked to provide a binary Yes/No or an ‘Increase/Decrease/No change’ answer. In the explanation tier students could choose one of the suggested explanations that best supported their reasoning underlying their answer. Except for the right explanation, all other explanations were designed to reflect possible misconceptions that students may hold. After the explanation tier, students had to answer the confidence tier: ‘How sure are you that your answer to the previous question was correct?’. Confidence was self-reported using a 5-pt Likert scale: 1: Very unsure (complete guess), 2: Fairly unsure, 3: In doubt, 4: Fairly sure, 5: Very sure (almost 100%). All questions were designed on the ‘apply’ and ‘analyse’ levels of Bloom’s taxonomy, and focused on examining students’ conceptual knowledge [40].

### Data analyses

IBM SPSS Statistics Version 23.0 (IBM Corp., Armonk, New York, USA) and GraphPad Prism Version 7.02 (GraphPad Software, La Jolla, California, USA) were used for all data analyses and visualizations. Descriptive statistics are provided as means and standard errors of the mean, unless otherwise mentioned. Only answers that consisted of a correct initial answer and a correct explanation were marked correct. Dependent samples t-tests were performed, for Refutation text and Standard text groups separately, to determine whether there was a difference in pre-test versus post-test scores.

An analysis of covariance (ANCOVA) was used to determine whether the post-test means, adjusted for pre-test scores, differed between groups. To determine the effects of response accuracy (incorrect or correct answer, i.e. cognitive effect), stage (pre- or post-intervention) and group (standard or refutation text), and their interactions with confidence (i.e. metacognitive effect), we used a multiple linear regression (MLR) model with dummy variables. We used effects coding to avoid multicollinearity. Consequently the coding for the dummy variables for response (R), stage (S) and group (G) was as follows: incorrect answer R = -1, correct answer R = + 1, pre-test S = -1, post-test S = + 1, standard text G = -1, refutation text G = + 1. The MLR model was: Y = B_0_ + B_R_.R + B_S_.S + B_G_.G + B_RS_.R.S + B_RG_.R.G + B_RSG_.R.S.G.

This model was applied to the individually corrected confidence scores (Y): a student’s average confidence score was subtracted from their confidence scores on each question to remove the between-students variability in average confidence scores.

**Fig. 1:**
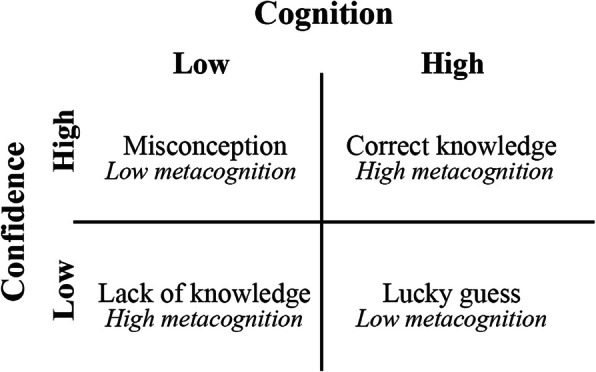
Hasan’s decision matrix (adjusted)

To test the hypercorrection hypothesis, we determined the fraction of initial misconceptions that were changed to a correct answer after intervention and the fraction of initial lack of knowledge that was changed to a correct answer. A hypercorrection effect is found if the fraction corrected misconceptions is higher than the fraction corrected lack of knowledge. Therefore, outcomes were made dichotomous: a confidence score below or equal to 3 was defined as low and a confidence score above 3 as high. This cut-off was chosen because students selecting “3” were still essentially unsure (‘in doubt’) about being correct. We used Hasan’s decision matrix to label the answers (see Fig. 1). According to this matrix incorrect answers given with high confidence are considered misconceptions, incorrect answers given with low confidence are considered a lack of knowledge [41]. Correct answers held with low confidence were labelled lucky guesses and correct answers with high confidence were labelled correct knowledge. In these terms, misconceptions and lucky guesses are considered low metacognition and correct knowledge and lack of knowledge high metacognition. Furthermore, a cognitive effect was labelled positive when one changed an incorrect answer to a correct answer. A metacognitive effect was labelled positive when one changed from low metacognition to high metacognition.

## Results

Table 1 presents the overall performance and average confidence scores on the pre-test and post-test in both groups. After reading Refutation texts, the overall test performance score increased significantly from 36.3% ± 0.03 to 58.8% ± 0.03 (t_(79)_ = 6976, *p* < 0.001). For the Standard text group a significant increase from 34.3% ± 0.03 to 57.1% ± 0.03 was found (t_(80)_ = 7198, *p* < 0.001). There was no significant difference in post-test performance between the group reading Refutation texts and the group reading Standard texts (F_(1,644)_ = 0.095, *p* = 0.758). The overall confidences scores increased significantly from 3.10 ± 0.06 to 3.52 ± 0.08 in the Refutation text group (t_(79)_ = 6154, *p* < 0.001). For the Standard text group a significant increase from 3.23 ± 0.06 to 3.58 ± 0.05 was found (t_(80)_ = 6101, *p* < 0.001). Additionally, there was also no significant difference in post-test confidence scores between the groups (F_(1,160)_ = 0.003, *p* = 0.954).

**Table 1:** Percentage correct of overall test performance and average confidence rating

	Refutation text (*n* = 80)	Standard text (*n* = 81)
Overall test performance (%)		
Pre test	36.3 ± 0.03	34.3 ± 0.03
Post test	58.8 ± 0.03	57.1 ± 0.03
Average confidence rating (max 5)		
Pre test	3.10 ± 0.06	3.23 ± 0.06
Post test	3.52 ± 0.08	3.58 ± 0.05

**Fig. 2:**
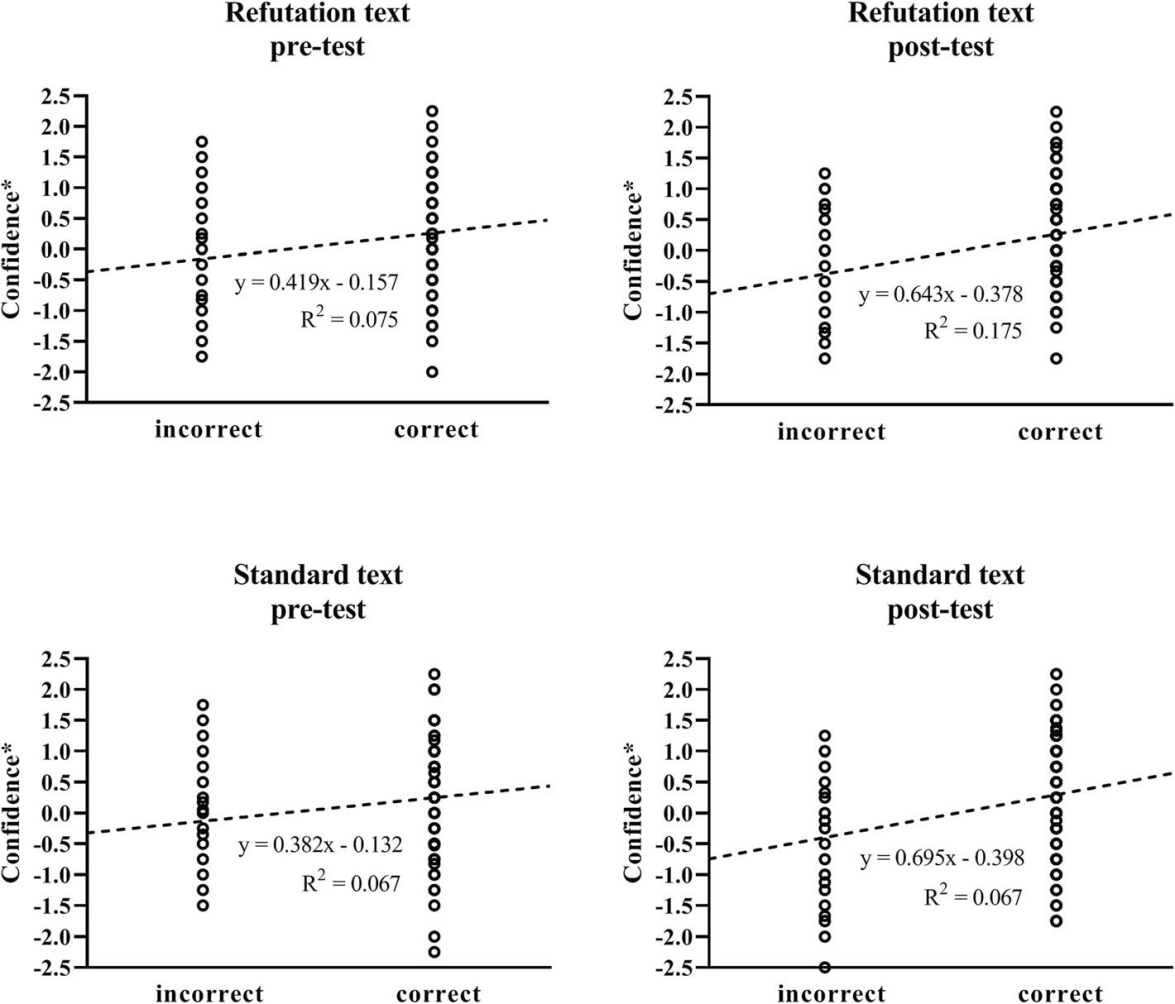
Relationship between students’ performance and confidence*

Figure 2 shows the relationship between students’ performance and confidence scores on individual questions. For each student, confidence scores per question were corrected for their average confidence to remove between-student variability in average confidence (i.e. confidence*, see Methods). In the refutation text group, the difference in confidence scores between incorrect and correct answers increased from Δ0.419 points pre-intervention to Δ0.643 points post-intervention. The standard text group showed a similar increase from Δ0.382 to Δ0.695 points. A complete overview of the numbers of incorrect and correct answers and related confidence and confidence* in both groups, at both stages is shown in Table 2.

**Table 2:** Number of incorrect and correct answers and related confidence and confidence^*^ scores (mean ± SD) in both groups, at both stages

Response (R)	Stage (S)	Group (G)	N	Confidence	Confidence^*^
Incorrect answer	Pre test	Standard text	214 (16.6%)	3.13 ± 0.84	−0.13 ± 0.59
		Refutation text	197 (15.3%)	2.92 ± 0.89	−0.16 ± 0.68
	Post test	Standard text	141 (10.9%)	3.14 ± 0.83	−0.40 ± 0.69
		Refutation text	131 (10.2%)	3.11 ± 0.96	−0.38 ± 0.61
Correct answer	Pre test	Standard text	113 (8.8%)	3.42 ± 0.99	0.25 ± 0.82
		Refutation text	116 (9.0%)	3.40 ± 0.91	0.26 ± 0.77
	Post test	Standard text	189 (14.7%)	3.91 ± 0.94	0.30 ± 0.82
		Refutation text	187 (14.5%)	3.80 ± 0.97	0.27 ± 0.74

Multiple linear regression analysis was used to determine main and interactive effects of response, stage and group on confidence*, see Table 3. Response and stage were significant predictors. The significant interaction effect between response and stage indicates that the difference in confidence* between incorrect and correct answers (i.e. the response effect) was significantly higher post-intervention than pre-intervention. This interaction effect, however, was not significantly different between the groups, as indicated by the lack of significance for the response-stage-group interaction term.

**Table 3:** Predictors of Confidence^*^

Parameter	B	Significance
Threshold	0.001	0.955
Response	0.267	0.000
Stage	−0.055	0.008
Group	−0.003	0.880
Response x Stage	0.067	0.001
Response x Group	−0.002	0.930
Stage x Group	0.00003	0.998
Response x Stage x Group	−0.011	0.585

Response; incorrect answer (−1), correct answer (+ 1). Stage; pre-test (− 1), post-test (+ 1). Group; standard text (− 1), refutation text (+ 1)

**Fig. 3:**
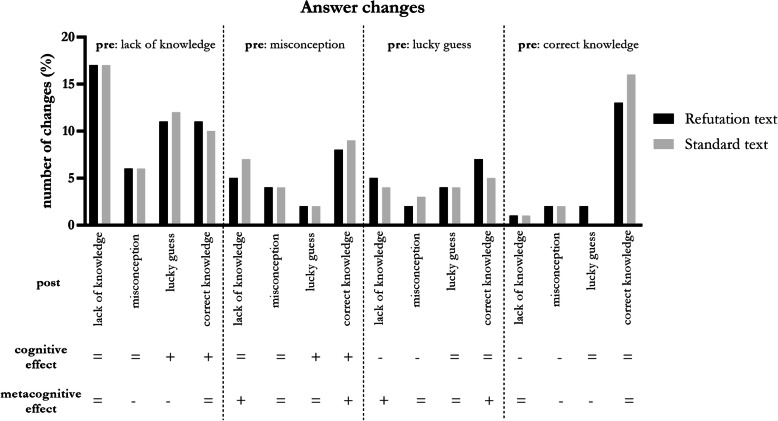
Answer changes on pre-post test

Figure 3 displays all changes, from pre- to post-intervention, in performance and confidence for the refutation and standard text groups. Both groups showed comparable changes. A positive *cognitive effect* was indicated if initially incorrect answers (i.e. misconception or lack of knowledge) were changed to correct answers (i.e. lucky guess or correct knowledge). A positive *metacognitive effect* was indicated if initially low metacognitive accuracy (i.e. misconception or lucky guess) changed to high metacognitive accuracy (i.e. lack of knowledge or correct knowledge). In the refutation text group, an overall positive cognitive effect was measured in 31.9% of cases compared to a negative cognitive effect of 9.4%. The positive metacognitive effect was 23.8% compared to a negative metacognitive effect of 21.3%. In the standard text group, the overall positive and negative cognitive effects were 32.5 and 9.0%, and the metacognitive effects were 25.0 and 20.5% respectively.

The hypercorrection hypothesis was tested by comparing the percentages of low versus high confidence incorrect answers that were changed to correct answers post-intervention. For the Refutation text group, 35.8% of the initially incorrect answers that were rated with high confidence (i.e. misconceptions) changed to correct knowledge after intervention. In contrast, initially incorrect answers rated with low confidence (i.e. lack of knowledge) were corrected to the right conception in 61.0% of cases. Similar findings were obtained in the Standard text group: the percentage of misconceptions that was corrected was 40.3% versus 66.0% of the lack of knowledge answers. Thus, these data do not support the hypercorrection hypothesis for either group. Rather, students with lack of knowledge more frequently corrected their answers than students with misconceptions.

## Discussion

With this study we investigated if reading refutation texts benefits conceptual understanding in medical students. Based on previous research we expected refutation texts to have a positive effect on students’ conceptual understanding (i.e. cognition) plus associated awareness of their understanding (i.e. metacognition). Additionally, based on the hypercorrection hypothesis we suggested that an increase in conceptual understanding would be present in students with high-confidence misconceptions in particular.

In summary, we found that reading refutation texts improved students’ cognition and metacognition but these effects were not significantly greater than the effects of reading standard texts. Furthermore, we could not find support for the hypercorrection hypothesis as students’ misconceptions were actually found harder to correct than correcting a lack of knowledge. Here, we elaborate on these findings and propose that instructional methods for concept learning should take into account the key role of metacognition, and the nature of misconceptions.

Since the cognitive and metacognitive improvements were found in both groups they could not be attributed to the refutation element, contrasting previous studies in higher education [19, 20]. Instead, the increase in both groups could be due to the answer and explanation elements that were present in both texts. According to the Knowledge Revision Components framework [13], co-activation of learners’ prior knowledge and new information may result in awareness of a possible cognitive conflict. Learners attempt to resolve this issue, leading to enhanced conceptual understanding. In our case, co-activation could have been induced by the answer or explanation element rather than the refutation element per se. Notably, many different misconceptions may be present among learners whereas the refutation element only addressed one of the supposedly most common misconceptions. Consequently, other possible alternative conceptions may have been left unaddressed, thereby limiting co-activation.

As indicated by an increase in accurate metacognitive judgements, co-activation seemed to be established to some extent in both groups, although, the absolute metacognitive outcomes remained relatively poor. These relatively poor metacognition scores align with findings from Thiede and colleagues reporting an average correlation of 0.27 between one’s actual performance and one’s self-perceived performance across 57 studies [42]. Since metacognition plays an important role in conceptual change processes, we suggest educators and researchers should pay more attention to the metacognitive component of learning [43]. This also relates to the view of the medical education community that students are expected to engage in their education as self-regulated learners [43, 44]. Self-regulated learning is an umbrella term that covers the cognitive, metacognitive, behavioural, motivational, and affective aspects of learning (for a review see Panadero, 2017) [45]. According to theory and practice, important metacognitive skills to facilitate self-regulated learning include planning, monitoring and evaluating. Optimizing these skills will contribute to effective learning, independent learning, and lifelong learning [46–48]. This comes with an important task for the medical educator as explicit teaching of these metacognitive skills inevitable; ‘learning how to learn cannot be left to students. It must be taught.’ [49–51].

The lack of additional benefit of the refutation element may be further explained by the nature of our misconceptions. As described by Chi, one can distinguish three types of knowledge representation: single ideas, mental models and categories [52]. Faulty ideas are suggested to be refuted more easily compared to flawed mental models or complex concepts such as physiological concepts. Regarding the latter, learners must generate inferences by connecting and understanding cause-effect relations [53, 54]. For our physiological misconceptions, refutation texts alone may not have been sufficient to achieve coherent concept representation. Additional educational approaches including diagramming, concept maps, problem-based learning and peer instruction may be needed to establish conceptual change for abstract scientific concepts [9, 55–57].

Contrary to the hypercorrection hypothesis, our findings showed that incorrect low confidence answers (i.e. lack of knowledge) were corrected more frequently than incorrect high confidence answers (i.e. misconceptions), after the interventions. This finding suggests that misconceptions are harder to correct than lack of knowledge which resonates with Conceptual Change Theory [15]. Again, the nature of misconceptions may play an important role in the ease with which conceptual change can be achieved. Interestingly, a previous study by van Loon et al. showed results similar to our study and suggested that the absence of the hypercorrection effect may also be clarified by the feedback format [29]. Both van Loon et al., and our study provided feedback (through text reading) to students on all questions, whether they held a misconception or not. Contrastingly, previous hypercorrection studies only provided feedback to learners when they made an error which might cause attentional bias towards the misconceptions [29]. Due to the contextual and protocol variations, it remains difficult to compare and generalise results across studies. Therefore, future studies in the specific context of medicine are needed to advance conceptual change research in medical education.

This research has limitations that need be considered when interpreting its results. Conceptual change is a gradual process, therefore, a longitudinal design including long-term outcomes may provide additional insights in students’ learning processes. Furthermore, our study was conducted in a real-life seminar setting and therefore comprised a limited number of questions. Additionally, we used a multi-tier approach with multiple choice answers [6]. Regarding students’ cognition, we cannot know if there were other alternative conceptions that were not explicitly stated in the assessment format. Regarding students’ metacognition, we cannot identify the metacognitive processes that occurred during reading as we only measured their confidence after reading. Future research may include open-ended questions or thinking aloud procedures to provide more information on students’ level of conceptual understanding and metacognitive processes.

## Conclusions

This study was the first to investigate the effect of refutation texts on conceptual understanding in medical students. Reading refutation texts did not significantly improve students’ cognition and metacognition beyond reading standard texts. Importantly, we found that misconceptions on cardiovascular physiology were robust and the accuracy of metacognitive judgements among medical students was relatively low. These findings have implications for classroom practice, by addressing the critical role of metacognition and the nature of misconceptions in physiological concept learning. Future studies should take into account these cognitive and metacognitive facets involved in students’ learning processes in order to develop effective teaching practices.

## Supplementary information

**Additional file 1.** Appendix A, Appendix B; A. Multi-tier question with 3-tiers: Yes/No, Explanation, and Confidence, B. Refutation text with a refutation element, correct answer, and explanation.

## Data Availability

The datasets used and analysed during the current study are available from the corresponding author on reasonable request.

## Abbreviations

ANCOVA: ANalysis of COVAriance
ERRB: Educational Research Review Board
G: Group
MLR: Multiple Linear Regression
R: Response
S: Stage
