# Supplementary material for: Refuting misconceptions in medical physiology
Source: BMC Med Educ. 2020 Aug 5;20:250. doi: 10.1186/s12909-020-02166-6 (PMC7409498; doi:10.1186/s12909-020-02166-6)
Supplement: Supplementary file 1 — Additional file 1. Appendix A, Appendix B; A. Multi-tier question with 3-tiers: Yes/No, Explanation, and Confidence, B. Refutation text with a refutation element, correct answer, and explanation. [file 12909_2020_2166_MOESM1_ESM.zip › Supplemental file (English)R1.docx]

**Appendix A.** Multi-tier question with 3-tiers: Yes/No, Explanation, and Confidence. *Note that all questions, answers, and explanation were translated in English for reading purposes. The original questions that were provided to the students were in Dutch.*

**
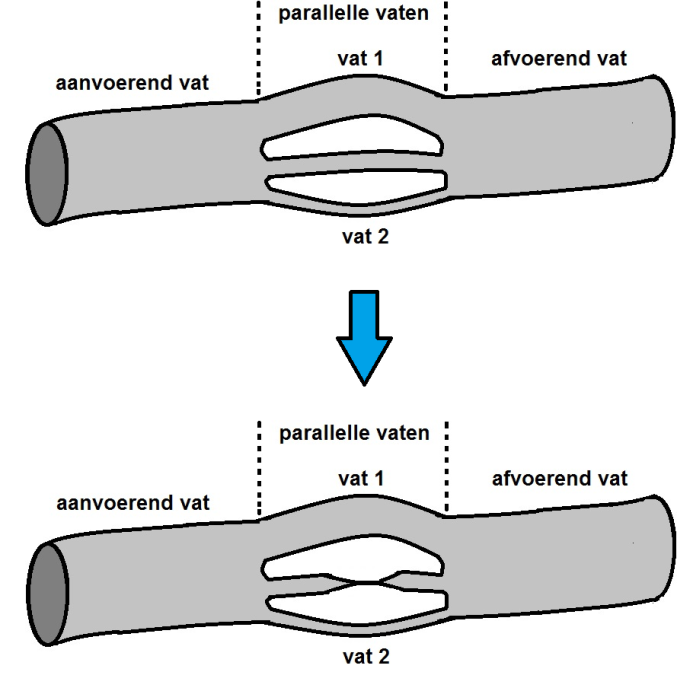
Question 2**

A surgeon observes a vessel structure in the leg of a patient: three parallel vessels with a feeding and draining vessel. The flow through vessel 1 is twice the flow through vessel 2.

The surgeon restricts the middle vessel, so no flow can pass through the middle vessel. The surgeon argues that the flow through vessel 1 is still twice the flow through vessel 2. You may assume that the diameters of vessel 1 and vessel 2 do not change.

MC: Is the surgeon right?

□ Yes

□ No

E: What is the best explanation you can provide for your answer?

□ The flow is divided over the vessels based on the ratio of resistance.

□ The flow will take the path of least resistance.

□ The total flow remains constant.

□ The total flow increases and thus the flow decreases equally in both vessels.

C: How sure are you that your given answer and explanation are both correct?

□ Very unsure (complete guess) □ Fairly unsure □ In Doubt □ Fairly sure □ Very sure (almost 100%)

**Appendix B.** Refutation text with a refutation element, correct answer, and explanation.

Some people say that the flow will always choose the path of least resistance, and thus will pass through vessel 1 in this case. That is incorrect, because flow does not always choose the path of least resistance, but it divides itself dependent on the individual resistance of each vessel. The vessel with the lowest resistance ‘gets’ most of the flow, but also a vessel with a higher resistance will ‘get’ flow.

The right answer is: Yes, the surgeon is right, because the flow is divided over the vessels based on the ratio of resistance.

The change in pressure is similar for all parallel vessels. Flow is pressure change divided by resistance, so the ratio of flows is determined by the ratio of resistances: the vessel with the lowest resistance ‘gets’ most of the flow. At the start, the flow in vessel 1 is twice the flow in vessel 2. Thus, apparently the resistance in vessel 1 is twice as low as the resistance in vessel 2. After the restriction of the middle vessel, the change in pressure may differ but this difference will still be the same for all vessels. The resistance of vessel 1 and 2 remain the same, so does their ratio. And thus, twice as much flow will pass through vessel 1 compared with vessel 2.

Maybe the flow through vessel 1 increases, but then that will also occur in vessel 2. However, the increase in vessel 2 will be half the increase from vessel 1, because the resistance in vessel 2 is twice as high as that from vessel 1. Although both flows may change, the ratio of these two flows will always be the same.
