# Supplementary material for: Refuting misconceptions in medical physiology
Source: BMC Med Educ. 2020 Aug 5;20:250. doi: 10.1186/s12909-020-02166-6 (PMC7409498; doi:10.1186/s12909-020-02166-6)
Supplement: Supplementary file 1 — Additional file 1. Appendix A, Appendix B; A. Multi-tier question with 3-tiers: Yes/No, Explanation, and Confidence, B. Refutation text with a refutation element, correct answer, and explanation. [file 12909_2020_2166_MOESM1_ESM.zip › Supplemental file (Dutch)R1.docx]

Appendix A.


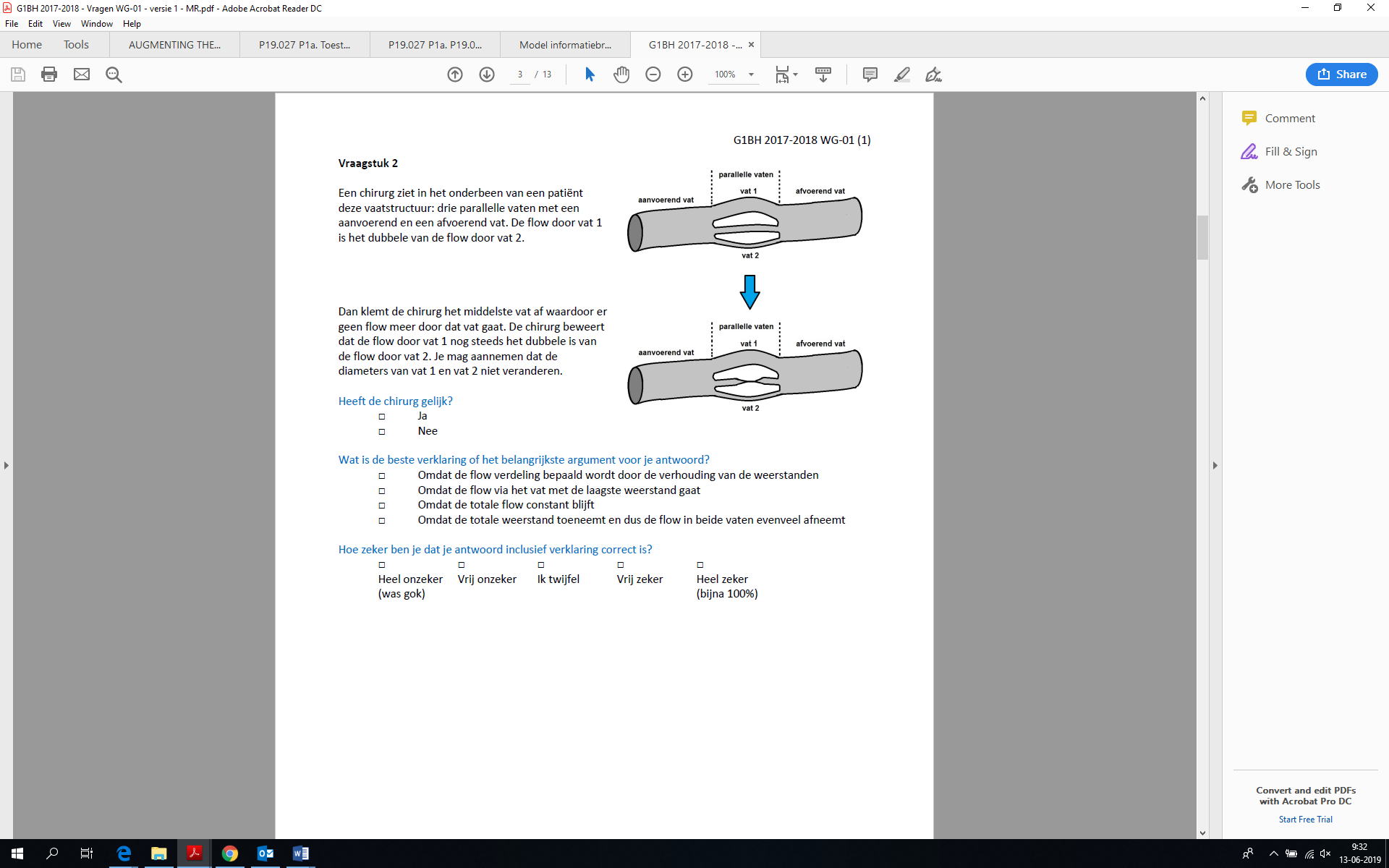


Multi-tier question (in Dutch) with 3-tiers: Yes/No, Explanation, and Confidence.

Appendix B.


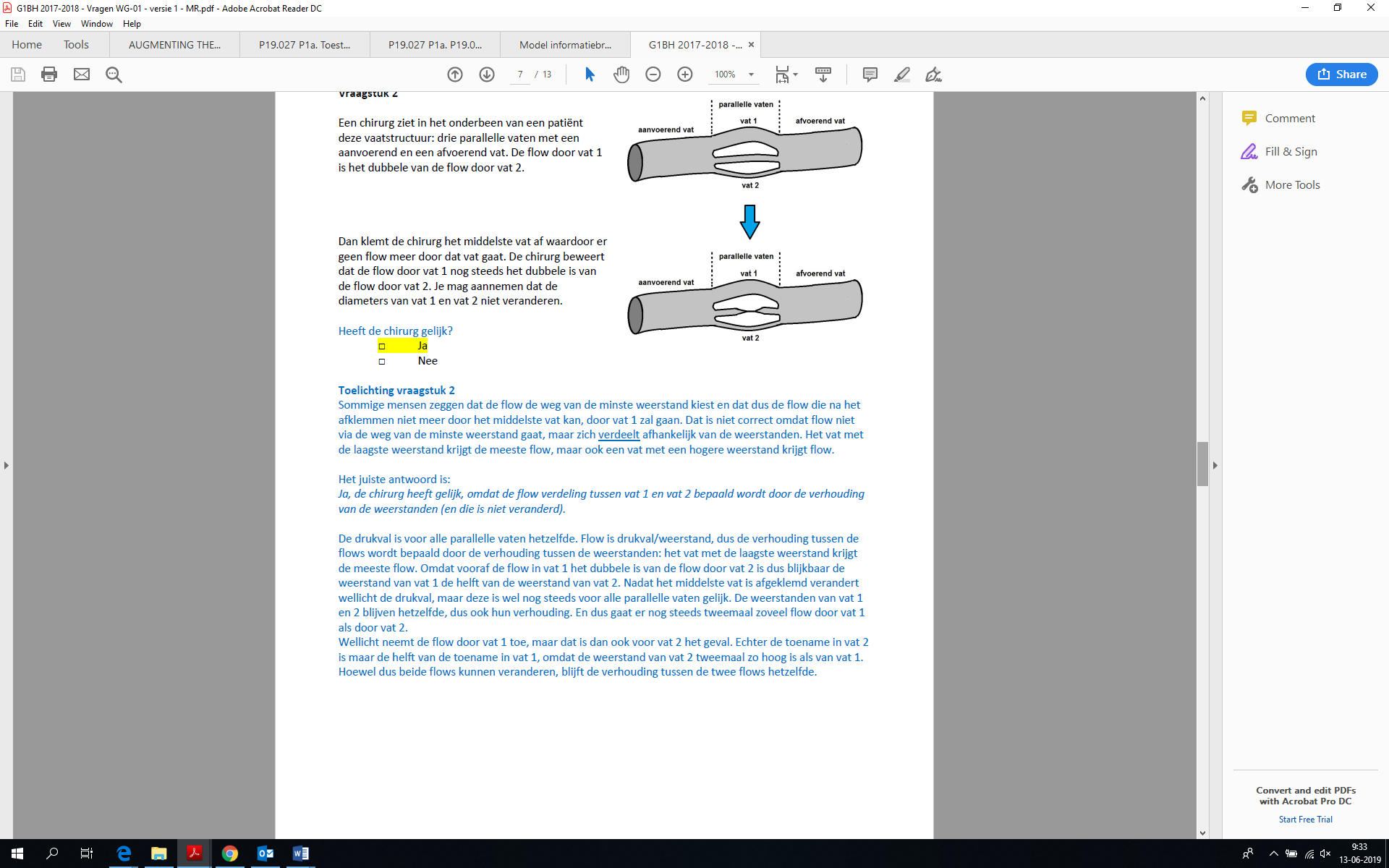


Refutation text (in Dutch) with a refutation element, correct answer, and explanation.
